# Neural correlates of cognitive bias modification for interpretation

**DOI:** 10.1093/scan/nsaa026

**Published:** 2020-04-23

**Authors:** Kohei Sakaki, Takayuki Nozawa, Shigeyuki Ikeda, Ryuta Kawashima

**Affiliations:** 1 Department of Functional Brain Imaging, Graduate School of Medicine, Tohoku University, Sendai 980-8575, Japan; 2 Division for Interdisciplinary Advanced Research and Education, Tohoku University, Sendai 980-8578, Japan; 3 Japan Society for the Promotion of Science, Tokyo 102-0083, Japan; 4 Research Institute for the Earth Inclusive Sensing Empathizing with Silent Voices, Tokyo Institute of Technology, Tokyo 152-8550, Japan; 5 Department of Ubiquitous Sensing, Institute of Development, Aging and Cancer, Tohoku University, Sendai 980-8575, Japan; 6 Department of Functional Brain Imaging, Institute of Development, Aging and Cancer, Tohoku University, Sendai 980-8575, Japan

**Keywords:** cognitive bias, social anxiety, positive thinking, social reward, fMRI

## Abstract

The effectiveness of cognitive bias modification for interpretation (CBM-I), a treatment method employed to reduce social anxiety (SA), has been examined. However, the neural correlates of CBM-I remain unclear, and we aimed to elucidate brain activities during intervention and activity changes associated with CBM-I effectiveness in a pre–post intervention comparison. Healthy participants divided into two groups (CBM, control) were scanned before, during and after intervention using functional magnetic resonance imaging. Ambiguous social situations followed by positive outcomes were repeatedly imagined by the CBM group during intervention, while half of the outcomes in the control group were negative. Whole-brain analysis revealed that activation of the somatomotor and somatosensory areas, occipital lobe, fusiform gyrus and thalamus during intervention was significantly greater in the CBM than in the control group. Furthermore, altered activities in the somatomotor and somatosensory areas, occipital lobe and posterior cingulate gyrus during interpreting ambiguous social situations showed a significant group × change in SA interaction. Our result suggests that when facing ambiguous social situations, positive imagery instilled by CBM-I is recalled, and interpretations are modified to contain social reward. These findings may help to suggest an alternative manner of enhancing CBM-I effectiveness from a cognitive-neuroscience perspective.

## Introduction

Although interpersonal relationships constitute a fundamental human motivation (Baumeister and Leary, 1995), we sometimes experience anxiety and a need to withdraw from social situations. It is not uncommon for people to fear negative evaluations and feel distress or discomfort in social situations (Watson and Friend, 1969). These feelings comprise social anxiety (SA), and it is assumed that SA exists on a wide spectrum, from shyness regarded as a general personality trait to SA disorder (SAD) requiring clinical support (Kashdan, 2007; Henderson *et al.,* 2014). Adolescents with higher levels of SA reported poorer social functioning, fewer friendships, and less intimacy, companionship, and support in their close friendships (La Greca and Lopez, 1998). Most university students, although comprising a nonclinical sample, experienced symptoms of SA such as sweating and shaking in their daily life (Purdon *et al.,* 2001). Therefore, SA reduction is an issue of great concern to the fields of psychiatry and education.

In the field of psychology, a cognitive model of SA has been investigated in detail (Mathews and MacLeod, 1994). A cognitive process that may mainly contribute to causality and maintain SA symptoms is interpretation bias (Rapee and Heimberg, 1997). Socially anxious individuals tend to interpret social stimuli and events in a negative manner even if the events and stimuli are emotionally ambiguous (Amin *et al.,* 1998; Hirsch and Mathews, 2000; Hirsch and Clark, 2004).

Cognitive bias modification for interpretation (CBM-I) is a training program aimed at reducing SA by modifying negative interpretation bias (Mathews and Mackintosh, 2000; Yiend *et al.,* 2005). In the typical CBM-I paradigm for SA, participants are requested to repeatedly mentally imagine presented series of social scenarios that are initially ambiguous and are followed by a clearly positive (or benign) interpretation outcome (MacLeod and Mathews, 2012). Initial CBM-I studies investigated the effectiveness of positive interpretation training to reduce trait anxiety in healthy populations (Mathews and MacLeod, 2002; Mathews *et al.,* 2007). Recent previous studies have reported that the effect of CBM-I modifying biased interpretation could transfer to reduce SA symptoms (Murphy *et al.,* 2007; Beard and Amir, 2008; Amir and Taylor, 2012; Bowler *et al.,* 2012; Khalili-Torghabeh *et al.,* 2014; Brettschneider *et al.,* 2015; Yeung and Sharpe, 2019). Although CBM-I has promising potential, meta-analyses have indicated that the effect size of CBM-I was small and its effectiveness should be enhanced (Hallion and Ruscio, 2011; Menne-Lothmann *et al.,* 2014; Cristea *et al.,* 2015; Liu *et al.,* 2017; Krebs *et al.,* 2018). Several clinical studies have attempted to overcome this challenge, boosting the effectiveness of CBM-I by combining it with other psychiatric treatments such as cognitive behavior therapy (CBT) (Butler *et al.,* 2015; Stevens *et al.,* 2018) or CBM for attention (Beard *et al.,* 2011; Naim *et al.,* 2018; Yeung and Sharpe, 2019). In addition to such clinical challenges, basic research for greater understanding of the mechanisms underpinning the effectiveness of CBM-I would contribute toward shedding light from different directions and lead to developing an innovative approach to improve CBM-I. Although several experimental studies and clinical trials have been accumulated, the underlying neural correlates of CBM-I remain understudied and unclear.

In general CBT using reappraisal, it is well-known that the dorsal medial and lateral prefrontal cortices play a key role in top–down emotion regulation (Simpson *et al.,* 2000; Ochsner *et al.,* 2002, 2012; Goldin *et al.,* 2008; Ochsner and Gross, 2008; Wager *et al.,* 2008). In contrast, CBM-I was assumed to have relatively implicit and automatic action mechanisms and was reported to work even under conditions when cognitive resources were depleted (Bowler *et al.,* 2012). Mathews (2012) proposed two potential CBM-I action mechanisms; one was semantic priming of a category of affective meanings and the other was transfer of a learned form of processing from training to testing. The affective priming effect is a phenomenon where processing or responses for target stimuli are facilitated by the prior presentation of a prime when they are affectively congruent (Fazio, 2001; Winkielman *et al.,* 2005). Applying this to the CBM-I context, positive outcomes repeatedly presented during intervention act as primes and facilitate the positive interpretation circuit when the participants face novel ambiguous social situations immediately after intervention. In fact, previous CBM-I studies supported that participants trained to make positive (or negative) interpretations by related affective priming homographs were faster to identify positive (or negative) words in a lexical decision task (Grey and Mathews, 2009; Hoppitt *et al.,* 2010b). Here, we hypothesized that the nucleus accumbens (NAcc) would play a key role in the affective priming underlying CBM-I. The NAcc is a core element of the subcortical reward circuitry included in the ventral striatum and activates in response to various reward stimuli such as money, food and drugs (Berridge, 2003). NAcc activation was related to affective priming using happy-face stimuli, which are known to be treated as ‘social reward’ by the brain (Winkielman *et al.,* 2007; Suslow *et al.,* 2013).

Conversely, only the affective priming effect is not sufficient to explain why modified interpretation could endure over longer periods (Mackintosh *et al.,* 2006) and transfer to novel stimuli that are not similar to those used in the training task (Hoppitt *et al.,* 2010b). Participants may learn a processing rule to select positive meanings following encountering ambiguity through training, and that rule would be unintentionally transferred and applied to subsequent events (Mathews and MacLeod, 2002). Rule learning through CBM-I could be successful under conditions that former parts of stimuli had affective ambiguity (Clarke *et al.,* 2014; Clifton *et al.,* 2016) and subsequent positive interpretations were actively selected (Hoppitt *et al.,* 2010a,b). However, totally self-generation of positive resolutions for ambiguous scenarios was not beneficial to enhance CBM-I effectiveness (Rohrbacher *et al.,* 2014). Additionally, presenting model answers or providing brief feedback would be required to reinforce the association between ambiguous social situations and subsequent positive interpretations (Murphy *et al.,* 2007; Beard and Amir, 2008; Bowler *et al.,* 2012). Based on this rule-learning hypothesis, we expected that brain areas related to memory functions would activate in response to learning rules for positive interpretations during intervention, and their activation would increase after, compared to before, intervention, reflecting retrieval of learned rules. It is well known that the hippocampus is involved in three successive processes comprising encoding, storage and retrieval (Squire, 1992; Burgess *et al.,* 2002). Additionally, the amygdala is associated with emotional learning and memory (Gallagher and Holland, 1994; Phelps, 2004, 2006; Phelps and LeDoux, 2005). The amygdala tends to become more highly active in response to negative, than to positive, emotions, but a previous study found that the amygdala was more active when more optimistic people, who tend to expect better outcomes, imagined future positive, compared to negative, events (Sharot *et al.,* 2007). Furthermore, the hippocampus and amygdala are associated with explicit and implicit memory (Rose *et al.,* 2002; Reber, 2013), associative learning (Killgore *et al.,* 2000) and affective conditioning (Büchel *et al.,* 1999).

Here, brain activities were examined using functional magnetic resonance imaging (fMRI) while participants imagined outcomes of ambiguous social situations before, during and after intervention to examine the neural correlates of CBM-I, which remains understudied. Interpretation bias, which was assessed by subjective ratings of expectation for novel ambiguous social situations, and SA, evaluated by a questionnaire, were assessed at pre- and post-intervention. To examine the neural correlates of CBM-I, we mainly used three types of analysis. First, we analyzed imaging data scanned during intervention to reveal the brain areas that were activated during CBM-I. Second, we analyzed the data derived from the assessment task to locate the brain areas whose activities would be altered by CBM-I. Third, multiple regression analysis using change in assessment scores was performed to search for brain areas reflecting individual differences in CBM-I effectiveness. Based on the review of the neuroimaging literature mentioned above, our hypotheses were that brain areas associated with social reward and emotional memory, such as the NAcc, hippocampus and amygdala, would be more activated during CBM-I compared to the control intervention, and activities in these areas during interpreting novel ambiguous social situations would increase after intervention. Moreover, participants with greater activity changes in these areas would show greater SA reduction.

## Material and methods

### Participants

A total of 40 healthy right-handed Japanese university students (20 men and 20 women, age range 20–26 years; mean, 22.0 years) participated in the experiment. The sample-size estimation was based on a previous study that investigated the effectiveness of mental imagery CBM-I using group comparisons (Clarke *et al.,* 2014) and was consistent with other relevant studies (Browning *et al.,* 2010; Taylor *et al.,* 2014; Beevers *et al.,* 2015; Britton *et al.,* 2015). Handedness was evaluated using the Edinburgh Handedness Inventory (Oldfield, 1971). No participant had history of neurological or psychiatric illness. The experimental protocol was approved by the ethics committee of the Tohoku University School of Medicine and conformed to the Declaration of Helsinki. Written informed consent was obtained from all participants.

Each of the two groups included the same number of participants (10 men and 10 women) who exhibited similar tendency toward SA. SA tendency was measured with the Japanese version of the fear of negative evaluation scale (FNE; Ishikawa *et al.,* 1992), and the participants were allocated so that they would be roughly matched between the two groups based on their score on the scale.

The data of two participants (one woman in the CBM group and one man in the positive–negative [PN] group) were excluded due to insufficient task performance (response rates <80%).

### Intervention task

**Fig. 1:**
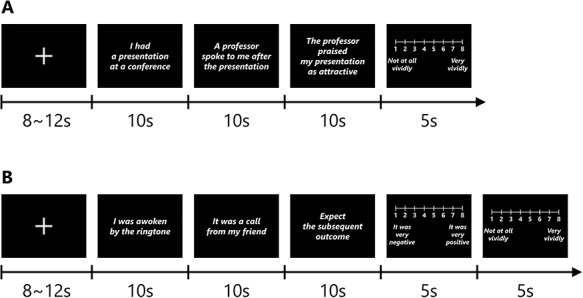
Schematic of the sequence of trial events in the intervention task (A) and the assessment task (B). (A) A small fixation cross was presented for 8–12 s as the ITI. Three short sequential sentences composing a single social scenario were presented for 10 s each, and participants imagined the situations. The initial two sentences denoted an ambiguous outcome and the third sentence denoted a positive (or negative) outcome. Participants rated the vividness of their imagery for the present scenario on an eight-point Likert scale from 1 (Not at all vividly) to 8 (Very vividly) within 5 s. (B) A small fixation cross was presented for 8–12 s as the ITI. Two short sequential sentences composing a single social scenario were presented for 10 s each, and participants imagined the situations. The two sentences denoted ambiguous outcome, and participants were required to expect the subsequent outcome for the present scenario and imagine it for 10 s. Participants rated the valence of their expected outcome on an eight-point Likert scale from 1 (It was very negative) to 8 (It was very positive) within 5 s and the vividness of their imagery for the present scenario on an eight-point Likert scale from 1 (Not at all vividly) to 8 (Very vividly) within 5 s.

We constructed an intervention task based on previous studies (Holmes and Mathews, 2005; Holmes *et al.,* 2006; Blackwell and Holmes, 2010; Clarke *et al.,* 2014). One hundred scenarios consisting of three short sentences were created (see Supplementary Data for details). All scenarios had to include some characters and be related to a daily social situation. Participants were instructed to imagine all scenarios as vividly as possible playing the role of the main character. Participants could not determine whether the end of the scenario was positive or negative simply by reading the two initial sentences (e.g. ‘I had a presentation at a conference/A professor spoke to me after the presentation’). All scenarios in the CBM group had a positive outcome (e.g. ‘The professor praised my presentation as attractive’). Conversely, half the scenarios in the PN group had a negative outcome (e.g. ‘The professor castigated my presentation as incomprehensible’). This manner of control intervention had no effect on interpretation bias (Salemink *et al.,* 2009, 2014). Every sentence was presented for 10 s on a screen in an MRI scanner. After three sentences were presented, the participants were asked to rate how vividly they could imagine the scenario on an eight-point Likert scale from 1 (*Not at all vividly*) to 8 (*Very vividly*) within 5 s. Afterward, a small fixation cross was presented for 8–12 s as the intertrial interval (ITI). The experimental design is illustrated in Figure 1A. One session consisted of 20 scenarios and lasted for ~15 min. The participants rested for 15 min after every session and completed five sessions.

### Assessment task for interpretation bias

We also constructed an assessment task for interpretation bias based on a previous study (Holmes *et al.,* 2006). Thirty scenarios consisting of three short sentences were created (see Supplementary Data for details). In the case of the assessment task, only the initial two sentences were presented for 10 s each. The participants were required to freely interpret the initial two sentences, create an outcome for the scenario and imagine it for 10 s. Then, participants rated how positive was the imagined outcome on an eight-point Likert scale from 1 (*It was very negative*) to 8 (*It was very positive*) within 5 s. We defined the interpretation bias as the average scores of the affective ratings in each session (pre and post the intervention sessions) for every participant. Moreover, they rated within 5 s how vividly they imagined the scenario. Afterward, a small fixation cross was presented for 8–12 s as the ITI. The experimental design is illustrated in Figure 1B. One session consisted of 15 scenarios and lasted for ~13 min. The participants performed one session of the assessment task before and after intervention. Two sets of scenarios were prepared and used with the order of the sets counterbalanced among participants.

### Assessment of SA

We focused on participant tendency toward SA as the first outcome of intervention. Participants completed the FNE (Watson and Friend, 1969) before and after intervention (see Supplementary Data for further details).

### Statistical analysis for behavioral and psychological data

Behavioral and psychological data were analyzed using SPSS (version 23; IBM, Armonk, NY, USA). We performed mixed-ANOVA including time (pre, post) as a within-subjects factor and group (CBM, PN) as a between-subjects factor for the SA and interpretation bias scores.

### fMRI data acquisition and preprocessing

Both experimental tasks were conducted in an MRI scanner (3T Philips Achieva, Best, Netherlands). Participants comfortably laid on the bed of the MRI scanner with their heads fixed in the head coil using elastic bands. The visual stimulus was back-projected onto a semi-lucent screen behind the head coil and was viewed via a mirror. The size of the visual stimuli in all tasks subtended a visual angle of less than 5° (see Supplementary Data for further details).

### Overview of fMRI data analysis

Both fMRI tasks were modeled using a block design. All imaging data were analyzed using a two-level approach in SPM12. At the first-level, the hemodynamic responses and the time derivatives generated by a participant under the different experimental conditions were assessed at each voxel using a general linear model. We constructed separate canonical regressors corresponding to each phase of the task in the trial, and the mean centered rating values as parametric regressors and confounding factors (head motion and magnetic field drift) were incorporated into the model. ITIs were not modeled. For second-level whole-brain analysis, the cluster-forming threshold at the voxel-level was set at *P* < 0.001 (uncorrected) and corrected for multiple comparisons at the cluster-level (family-wise error [FWE], *P* < 0.05, one-tailed).

### fMRI data analysis of the intervention task

For second-level analysis, we performed a two-sample *t*-test using contrast images corresponding to brain activities during the third sentence-imagining phase. From whole-brain analysis, we located the brain areas where activity in the CBM group was greater than that in the PN group. Additionally, based on the a priori hypothesis that brain areas associated with social reward and emotional memory would be more activated during CBM-I than during the control intervention, we applied small volume correction (SVC) using a region of interest (ROI) including the bilateral NAcc, hippocampus and amygdala as defined in the Wake Forest University PickAtlas (Maldjian *et al.,* 2003). For ROI analysis, the statistical threshold was set at *P* < 0.05 FWE-corrected (peak-level, one-tailed).

Furthermore, psychophysiological interaction (PPI) analyses were conducted to examine relations between differences in group conditions and functional connectivity of the bilateral NAcc, hippocampus and amygdala seed regions utilizing the generalized psychophysiological interaction (gPPI) toolbox (McLaren *et al.,* 2012). The cluster-forming threshold at the voxel-level was set at *P* < 0.001 (uncorrected) and corrected for multiple comparisons at the cluster-level (FWE, *P* < 0.016, one-tailed), as three statistical tests were performed for each seed region.

### fMRI data analysis of the assessment task

For second-level analysis, we performed a two-sample *t*-test using contrast images from the difference in brain activities during the interpretation phase between post and pre intervention (post−pre) within subjects and tested for group (CBM, PN) × time (pre, post) interaction on activity. To detect brain areas corresponding to the individual differences in CBM-I effectiveness, i.e. a significant target of this study, we performed voxel-wise multiple regression analysis using the contrast images (post−pre) and the change in SA (ΔSA) as covariates of interest. The groups were included in the same model to examine the group × ΔSA interaction on activity changes (see Supplementary Data for further details). Additionally, based on the a priori hypothesis that activities in brain areas associated with social reward and emotional memory during interpreting novel ambiguous social situations would increase after intervention specifically in the CBM group, we applied SVC using the ROI including the bilateral NAcc, hippocampus and amygdala. Furthermore, gPPI analyses were conducted to examine the group × ΔSA interaction on functional connectivity changes (ΔConnectivity) of the bilateral NAcc, hippocampus and amygdala seed regions.

**Table 1:** Summary of the behavioral ratings and scores on the psychological scales

Measure	CBM group (*N* = 19)			PN group (*N* = 19)		
	Pre, mean (s.d.)	Post, mean (s.d.)	Mean difference [95% CI]	Pre, mean (s.d.)	Post, mean (s.d.)	Mean difference [95% CI]
SA	15.42 (8.36)	13.21 (9.46)	−2.21 [−3.59, −0.83]	17.00 (9.15)	18.00 (9.92)	1.00 [−0.38, 2.38]
Interpretation bias	5.28 (0.56)	5.66 (0.91)	0.38 [0.02, 0.73]	5.39 (0.69)	5.16 (0.80)	−0.23 [−0.58, 0.13]
Vividness rating	5.25 (0.85)	5.54 (0.81)	0.29 [−0.06, 0.64]	5.77 (1.07)	5.72 (0.92)	−0.06 [−0.40, 0.29]

Note: SA: the average score of the FNE scale; CBM: cognitive bias modification; PN: positive–negative; interpretation bias: the average score of the positive ratings; vividness rating: the average score of the vividness ratings; 95% CI: 95% confidence intervals (Bonferroni adjusted).

**Fig. 2:**
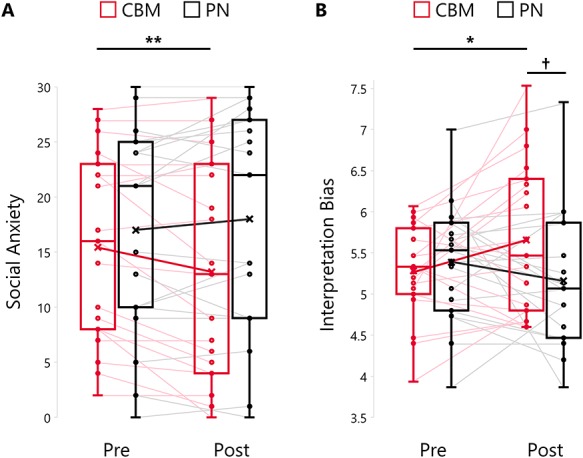
Distribution of scores of SA (A) and interpretation bias (B). Box plots show the median, interquartile range and minimum/maximum values of the scores for each group and session. (A) SA was assessed by the FNE. A high score on the FNE signifies high SA tendency. (B) Interpretation bias was calculated by the scores extracted from the assessment task. A high score of interpretation bias signifies the tendency of a participant to expect a positive outcome for ambiguous social situations. ^†^*P* < 0.1; ^*^P < 0.05; ^**^P < 0.01.

## Results

### Behavioral and psychological data

The summary of the behavioral ratings and scores on the psychological scales is presented in Table 1. A statistically significant interaction was revealed for SA (Figure 2A; *F*(1, 36) = 11.1, *P* = 0.002, η_p_^2^ = 0.24) and interpretation bias (Figure 2B; *F*(1, 36) = 5.89, *P* = 0.020, η_p_^2^ = 0.14). Additionally, *post-hoc* analysis with Bonferroni correction in the CBM group revealed a significant simple main effect of time (pre, post) for SA (*t*(18) = −3.09, *P* = 0.003, *r* = 0.59) and interpretation bias (*t*(18) = 2.08, *P* = 0.038, *r* = 0.44), which was not significant in the PN group (SA: *t*(18) = −1.55, *P* = 0.15, *r* = 0.34; interpretation bias: *t*(18) = 1.33, *P* = 0.21, *r* = 0.30).

### fMRI data

**Fig. 3:**
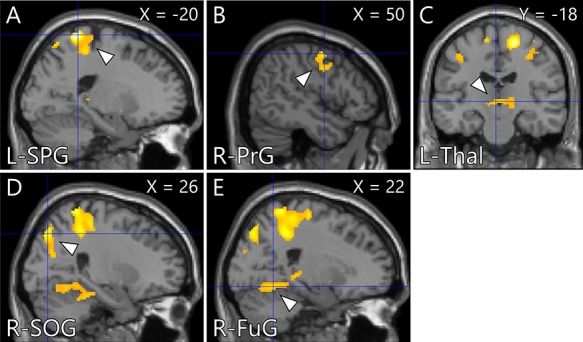
Brain areas showing greater activation in the CBM group than in the PN group during the intervention task. The cluster-forming threshold at the voxel level was set at *P* < 0.001 (uncorrected); multiple comparisons were corrected for at the cluster level (FWE, *P* < 0.05). (A) Left superior parietal lobule (−20, −44, 72). (B) Right PrG (50, −2, 52). (C) Left thalamus (−2, −18, 0). (D) Right SOG (26, −70, 22). (E) Right fusiform gyrus (22, −56, −12). CBM: cognitive bias modification; PN: positive-negative and FWE: family-wise error.

**Fig. 4:**
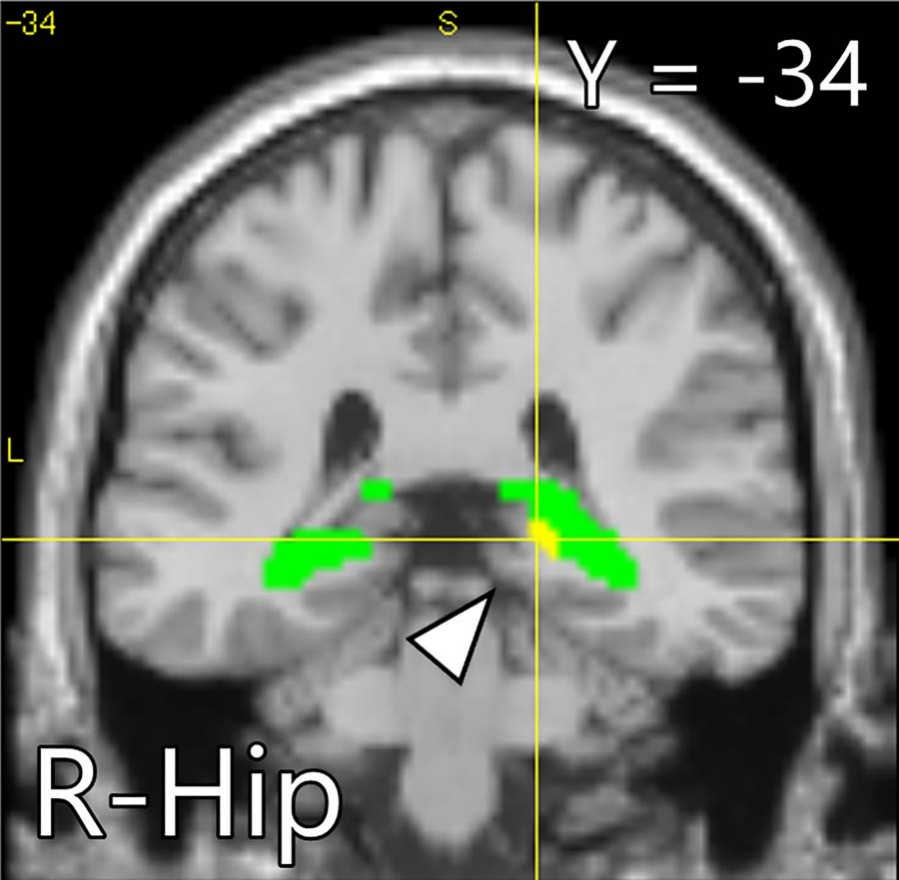
Brain areas showing greater activation (yellow) within the ROI (green) including the bilateral NAcc, hippocampus and amygdala in the CBM group than in the PN group during the intervention task. The statistical threshold at the voxel level was set at *P* < 0.001 (uncorrected) and corrected for SVC within the structural ROI at the peak level (FWE, *P* < 0.05). ROI: region on interest; CBM: cognitive bias modification; PN: positive-negative and FWE: family-wise error.

**Fig. 5:**
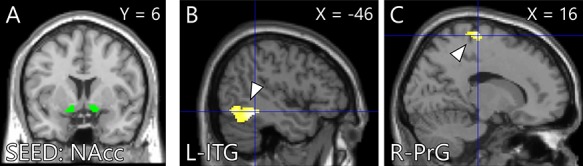
Brain areas showing greater functional connectivity with the bilateral NAcc seed region (A) in the CBM group than in the PN group during the intervention task. The cluster-forming threshold at the voxel level was set at *P* < 0.001 (uncorrected); multiple comparisons were corrected for at the cluster level (FWE, *P* < 0.016). (B) Left ITG (−46, −44, −12). (C) Right PrG (16, −24, 70). NAcc: nucleus accumbens; CBM: cognitive bias modification; PN: positive–negative and FWE: family-wise error.

Whole-brain analysis for the intervention task indicated that the activity of brain areas including the somatomotor and somatosensory areas, occipital lobe, fusiform gyrus (FuG) and thalamus in the CBM group was significantly greater than that in the PN group (Figure 3, Table 2). ROI analysis found that peak voxel activity in the right hippocampus was significantly greater in the CBM group than in the PN group (Figure 4, Table 3). The gPPI analysis revealed stronger connectivity between the bilateral NAcc and clusters located in the somatomotor and somatosensory areas, FuG and inferior temporal gyrus (ITG) in the CBM group than in the PN group (Figure 5, Table 4). The other seed regions (the hippocampus and amygdala) returned no significant results.

**Fig. 6:**
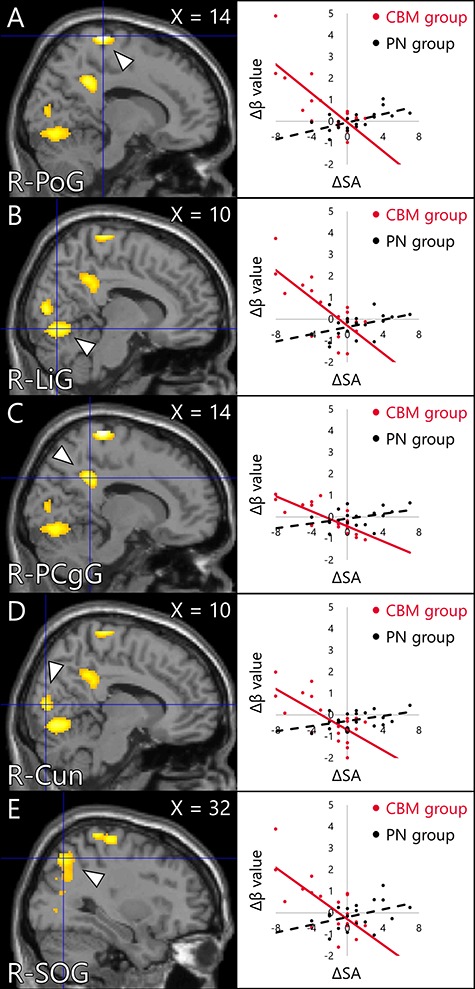
Brain areas in which the change in the amount of activity in the assessment task was associated with an interaction effect between the group and the change in SA. The cluster-forming threshold at the voxel level was set at *P* < 0.001 (uncorrected); multiple comparisons were corrected for at the cluster level (FWE, *P* < 0.05). (A) Right PoG (14, −32, 76). (B) Right lingual gyrus (10, −74, −10). (C) Right PCgG (14, −44, 34). (D) Right cuneus (10, −84, 8). (E) Right SOG (32, −68, 48). The Δβ values described in scatter plots were calculated by the difference of the peak voxel activity between pre and post intervention (post−pre) for each group (MIN = –1.99, MAX = 4.89). The ΔSAs were calculated by the difference of the FNE score between pre and post intervention (post−pre) for each group (MIN = –8, MAX = 7). FWE: family-wise error; SA: social anxiety and FNE: fear of negative evaluation.

**Fig. 7:**
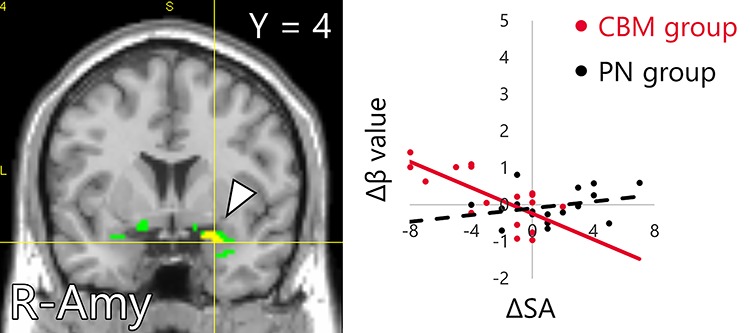
Brain areas in which the change in the amount of activity in the assessment task was associated with an interaction effect between the group and the change in SA (yellow) within the ROI including the bilateral NAcc, hippocampus and amygdala (green). The Δβ values described in scatter plots were calculated by the difference of the peak voxel activity between pre and post intervention (post−pre) for each group (MIN = –0.94, MAX = 1.44). The ΔSAs were calculated by the difference of the FNE score between pre and post intervention (post−pre) for each group (MIN = –8, MAX = 7). ROI: region of interest; SA: social anxiety and FNE: fear of negative evaluation.

**Fig. 8:**
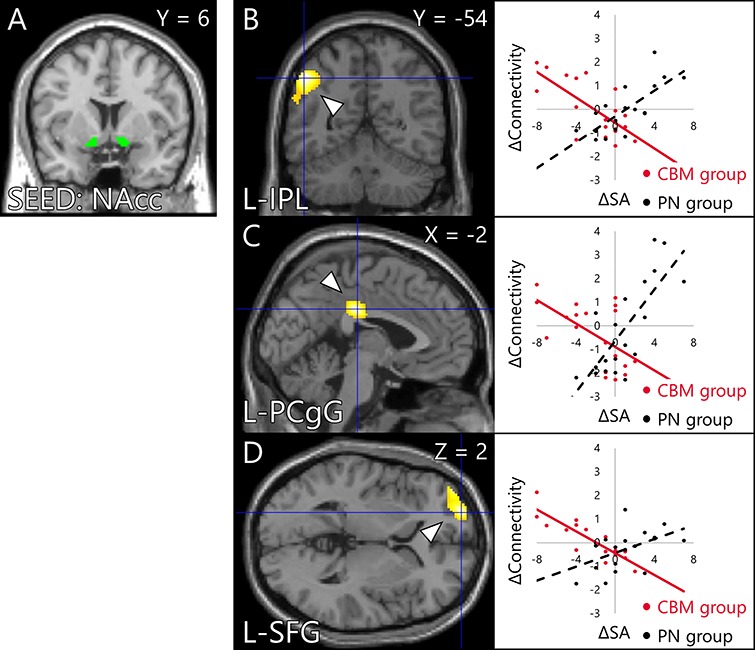
Brain areas in which the change in the amount of connectivity with the bilateral NAcc seed region (A) in the assessment task was associated with an interaction effect between the group and the change in SA. The cluster-forming threshold at the voxel level was set at *P* < 0.001 (uncorrected); multiple comparisons were corrected for at the cluster level (FWE, *P* < 0.016). (B) Left IPL (−50, −54, 44). (C) Left PCgG (−2, −24, 32). (D) Left SFG (−24, 62, 2). The connectivity changes (ΔConnectivity) described in scatter plots were calculated by the difference of the peak voxel connectivity between pre and post intervention (post−pre) for each group (MIN = –2.28, MAX = 3.65). The ΔSAs were calculated by the difference of the FNE score between pre and post intervention (post − pre) for each group (MIN = –8, MAX = 7). NAcc: nucleus accumbens; FWE: family-wise error; SA: social anxiety; and FNE: fear of negative evaluation.

The fMRI data of the assessment task indicated that there were no significant interaction of activity between the group (CBM, PN) and time (pre, post). The multiple regression analysis for the contrast images (post−pre) with the ΔSA as a regressor revealed a significant group × ΔSA interaction on activity changes in brain areas including the somatomotor and somatosensory areas, occipital lobe and posterior cingulate gyrus (PCgG) (Figure 6, Table 5). Part of areas including the somatomotor and somatosensory areas and superior occipital gyrus (SOG) overlapped with the activation area detected in the intervention task (Supplementary Figure S1). Focusing on the parametric regressors, the positive or vividness ratings modulated the activity of no brain area. ROI analysis found a significant group × ΔSA interaction on activity change in a peak voxel in the right amygdala (Figure 7, Table 6). The gPPI analysis revealed a significant group × ΔSA interaction on ΔConnectivity between the NAcc and clusters located in the inferior parietal lobule (IPL), PCgG and superior frontal gyrus (SFG) (Figure 8, Table 7). The other seed regions returned no significant results.

**Table 2:** Brain areas showing greater activation in the CBM group than in the PN group during the intervention task

Area		MNI peak coordinates (mm)				
		*x*	*y*	*z*	*t* value	*k*
Superior parietal lobule	L	-20	-44	72	6.49	4742
Supplementary motor area	R	18	-18	62	5.97	
	L	-12	-50	72	5.3	
PrG	R	50	-2	52	3.98	415
		48	-2	36	3.83	
PoG	R	56	0	42	3.67	
Thalamus	L	-2	-18	0	4.07	547
	R	14	-36	-4	3.98	
	L	-14	-30	2	3.96	
SOG	R	26	-76	44	4.79	322
		14	-86	34	4.00	
		26	-70	22	3.8	
Fusiform gyrus	R	22	-56	-12	4.00	405
		28	-34	-20	3.79	
		28	-48	-12	3.59	

Note: For each area, the coordinates (*x*, *y*, *z*) of the activation peak in MNI space, peak *t* value, and size of the activated cluster in a number (*k*) of voxels (2 × 2 × 2 mm^3^) are presented. The cluster-forming threshold at the voxel level was set at *P* < 0.001 (uncorrected) and corrected for multiple comparisons at the cluster level (FWE, *P* < 0.05). CBM: cognitive bias modification; PN: positive–negative; MNI: Montreal Neurological Institute and FWE: family-wise error.

**Table 3:** A peak voxel in the ROI showing greater activation in the CBM group than in the PN group during the intervention task

Area		MNI peak coordinates (mm)			
		*x*	*y*	*z*	*t* value
Hippocampus	R	18	-34	0	3.93

Note: For each voxel, the coordinates (*x*, *y*, *z*) in MNI space and *t* value are presented. The statistical threshold at the voxel level was set at *P* < 0.001 (uncorrected) and corrected for SVC within the structural ROI at the peak level (FWE, *P* < 0.05). ROI: region on interest; CBM: cognitive bias modification; PN: positive–negative; MNI: Montreal Neurological Institute and FWE: family-wise error.

**Table 4:** Brain areas showing greater functional connectivity with the bilateral NAcc seed region in the CBM group than in the PN group during the intervention task

Area		MNI peak coordinates (mm)				
		*x*	*y*	*z*	*t* value	*k*
ITG	L	-46	-44	-12	4.90	668
Fusiform gyrus	L	-18	-88	-10	4.69	
Inferior occipital gyrus	L	-34	-82	-10	4.52	
PrG	R	16	-24	70	4.77	673
PoG	L	-14	-38	72	4.68	
Paracentral lobule		0	-36	70	4.37	

Note: For each area, the coordinates (*x*, *y*, *z*) of the activation peak in MNI space, peak *t* value, and size of the activated cluster in a number (*k*) of voxels (2 × 2 × 2 mm^3^) are presented. The cluster-forming threshold at the voxel level was set at *P* < 0.001 (uncorrected) and corrected for multiple comparisons at the cluster level (FWE, *P* < 0.05). NAcc: nucleus accumbens; CBM: cognitive bias modification; PN: positive–negative; MNI: Montreal Neurological Institute and FWE: family-wise error.

**Table 5:** Brain areas in which the change in the amount of activity in the assessment task was associated with an interaction effect between the group and the change in SA

Area		MNI peak coordinates (mm)				
		*x*	*y*	*z*	*t* value	*k*
PoG	R	14	-32	76	6.35	787
		26	-36	72	5.84	
PrG	R	30	-24	66	4.64	
Lingual gyrus	R	10	-74	-10	5.02	1100
	L	-12	-76	-14	4.22	
Cerebellum	R	2	-72	-32	3.94	
PCgG	R	14	-44	34	4.97	359
Cuneus	R	10	-84	8	4.71	365
	L	-10	-76	14	4.61	
	R	4	-72	16	3.50	
SOG	R	32	-68	48	4.62	746
		24	-62	34	4.32	
		26	-76	18	3.92	

Note: For each area, the coordinates (*x*, *y*, *z*) of the activation peak in MNI space, peak *t* value, and size of the activated cluster in a number (*k*) of voxels (2 × 2 × 2 mm^3^) are presented. The cluster-forming threshold at the voxel level was set at *P* < 0.001 (uncorrected) and corrected for multiple comparisons at the cluster level (FWE, *P* < 0.05). MNI: Montreal Neurological Institute and FWE: family-wise error.

**Table 6:** A peak voxel in the ROI in which the change in the amount of activity in the assessment task was associated with an interaction effect between the group and the change in SA

Area		MNI peak coordinates (mm)			
		*x*	*y*	*z*	*t* value
Amygdala	R	24	4	-20	3.74

Note: For each area, the coordinates (*x*, *y*, *z*) of the activation peak in MNI space, peak *t* value, and size of the activated cluster in a number (*k*) of voxels (2 × 2 × 2 mm^3^) are presented. The statistical threshold at the voxel level was set at *P* < 0.001 (uncorrected) and corrected for multiple comparisons at the cluster level (FWE, *P* < 0.05). ROI: region of interest; MNI: Montreal Neurological Institute and FWE: family-wise error.

**Table 7:** Brain areas in which the change in the amount of connectivity with the bilateral NAcc seed region in the assessment task was associated with an interaction effect between the group and the change in SA

Area		MNI peak coordinates (mm)				
		*x*	*y*	*z*	*t* value	*k*
IPL	L	-50	-54	44	6.06	1106
Angular gyrus	L	-44	-68	36	5.05	
Supramarginal gyrus	L	-56	-50	28	3.92	
PCgG	L	-2	-24	32	5.80	516
		-14	-20	32	4.49	
SFG	L	-24	62	2	5.65	481
Middle frontal gyrus	L	-40	56	0	5.02	
SFG	L	-12	70	8	4.17	

Note: For each area, the coordinates (*x*, *y*, *z*) of the activation peak in MNI space, peak *t* value, and size of the activated cluster in a number (*k*) of voxels (2 × 2 × 2 mm^3^) are presented. The cluster-forming threshold at the voxel level was set at *P* < 0.001 (uncorrected) and corrected for multiple comparisons at the cluster level (FWE, *P* < 0.05). NAcc: nucleus accumbens; MNI: Montreal Neurological Institute and FWE: family-wise error.

## Discussion

To our knowledge, this study is the first to investigate neural correlates associated with CBM-I. We found greater brain activation in the somatomotor and somatosensory areas, occipital lobe, FuG and thalamus during CBM-I than during the control intervention. Stronger functional connectivity of the somatomotor and somatosensory areas, FuG and ITG with the NAcc was also found. Regarding brain activity changes, we found a group × ΔSA interaction on activity changes in the somatomotor and somatosensory areas, occipital lobe and PCgG during interpreting ambiguous social situations. Interaction on functional connectivity of the IPL, PCgG and SFG with the NAcc was also found.

### Neural correlates of CBM-I

Here, the activity in the somatomotor and somatosensory areas, occipital lobe, FuG and thalamus during the intervention task was significantly greater in the CBM than in the PN group. The FuG is known as the ‘face area’ (Rossion *et al.,* 2003; Kanwisher and Yovel, 2006) and becomes active in response to perception of happy facial expressions (Fusar-Poli *et al.,* 2009). The following structures are supplementally involved in response to happy facial stimuli: the superior parietal lobule, which plays a key role in various sensory and cognitive processes, including attention shifts (Corbetta *et al.,* 1995), sensorimotor integration (Wolpert *et al.,* 1998), visuomotor control (Culham *et al.,* 2006), mental rotation (Murphy, 2003) and working memory (Koenigs *et al.,* 2009); the thalamus that relays information between different subcortical areas and the cerebral cortex (Sherman and Guillery, 1996), and the SOG (Morris *et al.,* 1998; Lee *et al.,* 2002; Sato *et al.,* 2004). The precentral gyrus (PrG) and postcentral gyrus (PoG), which include the primary somatosensory cortex (Penfield and Rasmussen, 1950), activate in response to words and pictures bearing positive meaning (Kensinger and Schacter, 2006). The participants in the CBM group repeatedly imagined social situations ending consistently positively during the intervention task. The greater activation in the somatomotor and somatosensory areas, occipital lobe, FuG and thalamus possibly reflects the greater number of positive feelings induced by the happy faces and actions of the characters in the social situations that the participants repeatedly imagined during the task.

ROI analysis found that the hippocampus showed greater activation in the CBM group. The hippocampus was reported to be more active during memory encoding of pleasant stimuli than of neutral stimuli (Hamann *et al.,* 1999; Bulganin and Wittmann, 2015). Contrary to our initial hypothesis, the greater activation in the NAcc and amygdala was not significant. Moreover, gPPI analysis found stronger functional connectivity between the NAcc and the somatomotor and somatosensory areas, FuG and ITG in the CBM group. Numerous neuropsychological studies have shown that the NAcc is associated with various social reward such as happy and attractive faces (Cloutier *et al.,* 2008; Spreckelmeyer *et al.,* 2009; Mende-Siedlecki *et al.,* 2013), social approval and avoidance of social punishment (Kohls *et al.,* 2013), acquiring a positive reputation of oneself (Izuma *et al.,* 2008), social approach (Radke *et al.,* 2016), and prosocial behavior (van der Meulen *et al.*, 2016). All these types of social reward were possibly evoked during the intervention task. The stronger connectivity of the NAcc with the somatomotor and somatosensory areas and FuG also possibly supported the notion that the greater number of imageries in the CBM group was processed as social reward. Here, significant activity or functional connectivity differences between groups in the intervention task were not noted in the dorsal medial or lateral prefrontal cortex in line with previous findings.

### Brain activity changes induced by CBM-I

Here, the whole-brain analysis revealed a significant group × ΔSA interaction on activity changes in several brain areas including the somatomotor and somatosensory areas, occipital lobe and PCgG, observed while participants interpreted novel social situations in the assessment tasks. Furthermore, ROI analysis also revealed a group × ΔSA interaction on the activity change in the right amygdala. To wit, participants whose activity in these brain areas were further increased showed greater SA reduction after the CBM-I intervention. Moreover, gPPI analysis found a significant group × ΔSA interaction on ΔConnectivity between the NAcc and the IPL, PCgG and SFG.

These activity changes in the somatomotor and somatosensory areas including the PoG and PrG, which were also observed in the intervention task (Supplementary Figure S1A and B), possibly reflect increase in the number of happy faces and actions imagined in the novel social situations during the assessment task. The occipital lobe, which was also observed in the intervention task (Supplementary Figure S1C), including the SOG and cuneus forming the visual cortex, showed greater activity in response to emotional stimuli (Lang *et al.,* 1998; Park *et al.,* 2010). The greater activity increase in the visual cortex possibly reflects that participants could imagine more pleasant interpretations, which resulted in more positive emotional valence. In the PN group, this activity increase was not correlated with SA reduction because the emotional valence of social scenarios presented during the intervention task was not consistent. The NAcc and DLPFC are involved in reward prediction and motivation for goal-directed behavior (Goto and Grace, 2005; Knutson and Cooper, 2005; Watanabe and Sakagami, 2007; Ballard *et al.,* 2011). The activity increase in the visual cortex and reinforced functional connectivity between the NAcc and DLPFC that correlated with SA reduction also possibly supported the notion that the participants’ imagery was modified to be processed as higher social reward.

The IPL is associated with memory retrieval (Wagner *et al.,* 2005; Vilberg and Rugg, 2008) and working memory (LaBar *et al.,* 1999). The PCgG is also implicated in memory (Maddock *et al.,* 2001) and self-referential thinking (Johnson *et al.,* 2002; Kelley *et al.,* 2002; Northoff and Bermpohl, 2004; Northoff *et al.,* 2006). The IPL and PCgG are involved in the retrieval process particularly for self-referential episodic memory (Lou *et al.,* 2004). The activity increase in these memory-related areas and reinforced functional connectivity with the NAcc that correlated with SA reduction possibly reflects that participants attempted to self-referentially retrieve and recall the memory of positive interpretations for ambiguous social situations, learned during the intervention task. Furthermore, the activation increase in the amygdala might suggest that the participants who were more affected by intervention and experienced further reduction in SA became more optimistic as also shown by a previous study (Sharot *et al.,* 2007).

### Study implications

Although not directly supported by the present findings, we could provide some suggestions to improve the CBM-I protocol. To maximize the effect of CBM-I, experimenters should attempt to create stimuli that could be easily imagined vividly to further activate brain areas related to imagining, such as the face area, occipital lobe and somatomotor and somatosensory areas and induce highly positive emotions to further activate the NAcc and be strongly impressed in the memory of participants to activate brain areas involved in memory functions such as the hippocampus, amygdala, IPL and PCgG. Moreover, the present findings provided from the field of cognitive neuroscience could propose an alternative manner of enhancing effectiveness of CBM-I as a future direction. For example, neurofeedback, which also has an automatic and implicit action mechanism, could be well combined with CBM-I. Neurofeedback is a technique providing feedback on the brain activity of any ROI in real time as sensory input and self-regulating brain activity by reference to the feedback with or without explicit instructions (Heinrich *et al.,* 2007; Sulzer *et al.,* 2013; Stoeckel *et al.,* 2014; Scharnowski and Weiskopf, 2015). Based on the present findings, if brain activity (e.g. in the NAcc, hippocampus and amygdala) could be presented as feedback to participants during intervention, they could attempt to imagine social situations that could result in further activation in the relevant brain area. Thus, effectiveness of CBM-I could possibly be enhanced. In this manner, the present findings could both elucidate the neural correlates of CBM-I and lead to future studies aiming to improve and develop the intervention protocol for CBM-I.

### Limitations

University students that did not comprise a clinical sample were recruited. Further investigations are required to confirm whether the present results could be generalized to clinical populations or younger/older participants. For example, 12 20-min CBM-I intervention sessions delivered over 6 weeks were conducted in a previous randomized control trial that treated a clinical sample with SAD (Amir and Taylor, 2012). In another previous study, children with a diagnosis of SAD completed three 30-min sessions within 2 weeks (Orchard *et al.,* 2017). In this case, we constructed an intervention protocol that required participants to complete five 15-min sessions in a day with the aim to investigate acute effect of CBM-I. However, we would have to revise the intervention protocol following previous trials to reduce the burden of treatment on participants when especially patients or children would participate in the experiments. Moreover, in terms of neural correlates, previous neuroimaging studies have suggested that there were differences in widespread brain activities, connectivity and structures of patients with SAD compared to healthy controls (Bruhl *et al.,* 2014). Additionally, we should conduct follow-up assessments in a future study to confirm the long-term and far-transfer effects of treatment.

We expected that the NAcc, hippocampus and amygdala would be specifically associated with the CBM-I intervention and activities in these areas would increase after intervention. However, activities in these areas were no longer found to be significant in the whole-brain analysis for both the intervention and assessment tasks, and even the ROI analysis only partially returned significant results. This is the first study to address the neural correlates of CBM-I, and we focused on the acute effect of short-term intervention. It would be expected that longer-term intervention would show that the psychological and neurological acute effects verified here could accumulate and lead to more significant results. Furthermore, future research needs to investigate in detail the association of brain areas clarified here with specific potential action mechanisms of CBM-I such as affective priming and rule-learning.

The present experimental design could not completely exclude several confounding factors regarding the intervention task that potentially offer alternative explanations for the present results. For example, differences between the groups regarding the degrees of arousal, familiarity or movement and changes in mood or emotional states with the SA reduction may have influenced the differences in brain activities. Such potential confounding factors within the experimental and control conditions should be carefully removed to the extent possible in a future study (see Supplementary Data for further details).

## Conclusion

We identified brain areas associated with CBM-I and the effectiveness of CBM-I. Brain activation during CBM-I was greater in the somatomotor and somatosensory areas, occipital cortex, FuG and thalamus compared with the control intervention. Moreover, functional connectivity of the somatomotor and somatosensory areas, FuG and ITG with the NAcc was also stronger. This finding suggested that CBM-I activated the brain areas related to social reward perception and imagery of happy faces and actions via consistently and repeatedly imagining positive interpretations for ambiguous social situations. Regarding individual differences in intervention effectiveness, activity increase in the somatomotor and somatosensory areas, occipital lobe and PCgG during interpreting ambiguous social situations was associated with SA reduction specifically in the CBM group. Reinforced functional connectivity of the IPL, PCgG and SFG with the NAcc was also significantly correlated with SA reduction. This finding suggested that CBM-I effectiveness for SA reduction possibly reflected that memories of positive interpretations for ambiguous social situations, which were repeatedly instilled during the CBM-I intervention, was self-referentially retrieved and recalled, and imagery was modified to contain more social reward.

## Supplementary Material

File017_nsaa026
