# Supplementary material for: Neural correlates of cognitive bias modification for interpretation
Source: Soc Cogn Affect Neurosci. 2020 Apr 23;15(2):247–60. doi: 10.1093/scan/nsaa026 (PMC7304515; doi:10.1093/scan/nsaa026)
Supplement: File017_nsaa026 [file file017_nsaa026.doc]

SUPPLEMENTARY INFORMATION

Manuscript title:

Neural Correlates of Cognitive Bias Modification for Interpretation

Authors

Kohei Sakaki, Takayuki Nozawa, Shigeyuki Ikeda and Ryuta Kawashima

SUPPLEMENTARY TABLES

Table S1. Brain areas showing greater activation in the CBM group than in the PN group during the intervention task in the case of analyzing only positive trials in the PN group.

| Area | | MNI peak coordinates (mm) | | | | |
| --- | --- | --- | --- | --- | --- | --- |
|  |  | x | y | z | t value | k |
| Superior parietal lobule | L | -20 | -46 | 72 | 5.45 | 2154 |
|  | R | 14 | -54 | 68 | 4.81 |  |
| Precentral gyrus | R | 18 | -20 | 64 | 4.63 |  |
| Precentral gyrus | R | 42 | 0 | 48 | 4.11 | 488 |
|  |  | 56 | -2 | 40 | 3.90 |  |
|  |  | 54 | -4 | 48 | 3.82 |  |
| Thalamus | R | 16 | -20 | -6 | 4.27 | 398 |
|  | L | -2 | -16 | 0 | 4.03 |  |
|  | R | 20 | -30 | 2 | 3.88 |  |
| Putamen | L | -26 | -6 | -8 | 5.34 | 346 |
|  |  | -30 | -18 | -4 | 4.00 |  |

For each area, the coordinates (x, y, z) of the activation peak in Montreal Neurological Institute space, peak t value, and size of the activated cluster in a number (k) of voxels (2×2×2 mm^3^) are presented. The statistical threshold at the voxel level was set at p<0.001 and corrected for multiple comparisons at the cluster level (family-wise error, p<0.05). CBM: cognitive bias modification, PN: positive-negative

FIGURE LEGENDS

Fig. S1. Overlap between the brain areas that were detected in the intervention task and the assessment task. Red clusters represent brain areas that showed greater activation in the CBM group than in the PN group during the intervention task (Fig. 3, Table 2). Blue clusters represent brain areas wherein a positive correlation was noted between the amount of change in activity owing to intervention and reduction in the score of the Fear of Negative Evaluation (FNE) scale in the CBM group for the assessment task (Fig. 6, Table 4). Common parts of the red and blue areas are colored yellow. (A) Right precentral gyrus (38, -24, 54). (B) Right postcentral gyrus (16, -32, 74). (C) Right superior occipital gyrus (26, -72, 34). CBM: cognitive bias modification, PN: positive-negative

Fig. S2. Brain areas showing greater activation in the CBM group than in the PN group during the intervention task in the case of analyzing only positive trials in the PN group. The statistical threshold at the voxel level was set at p<0.001; multiple comparisons were corrected for at the cluster level (family-wise error, p<0.05). (A) Left superior parietal lobule (-20, -46, 72). (B) Right precentral gyrus (42, 0, 48). (C) Right thalamus (16, -20, -6). (D) Left putamen (-26, -6, -8). CBM: cognitive bias modification, PN: positive-negative

SUPPLEMENTARY FIGURES


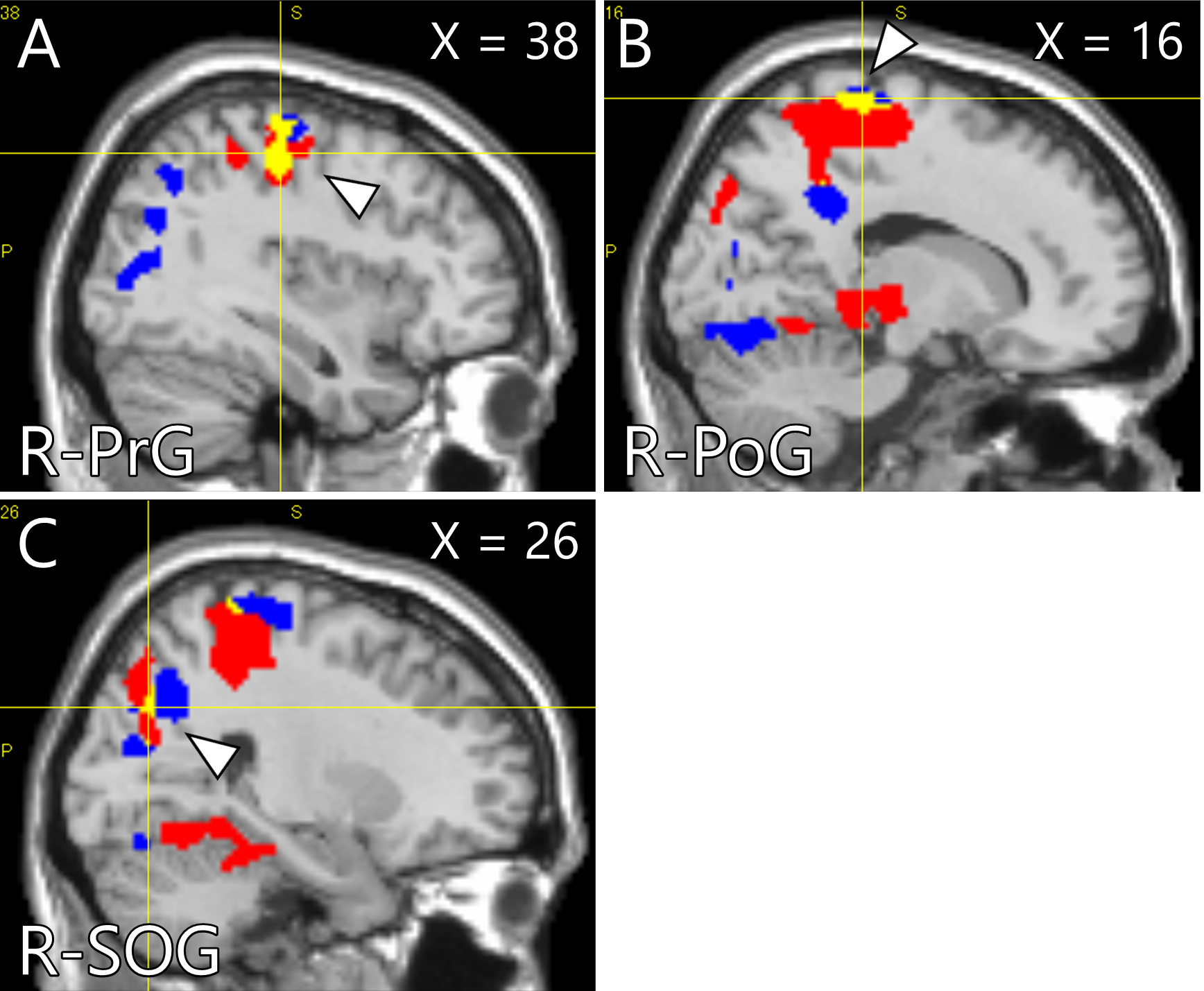
Fig. S1


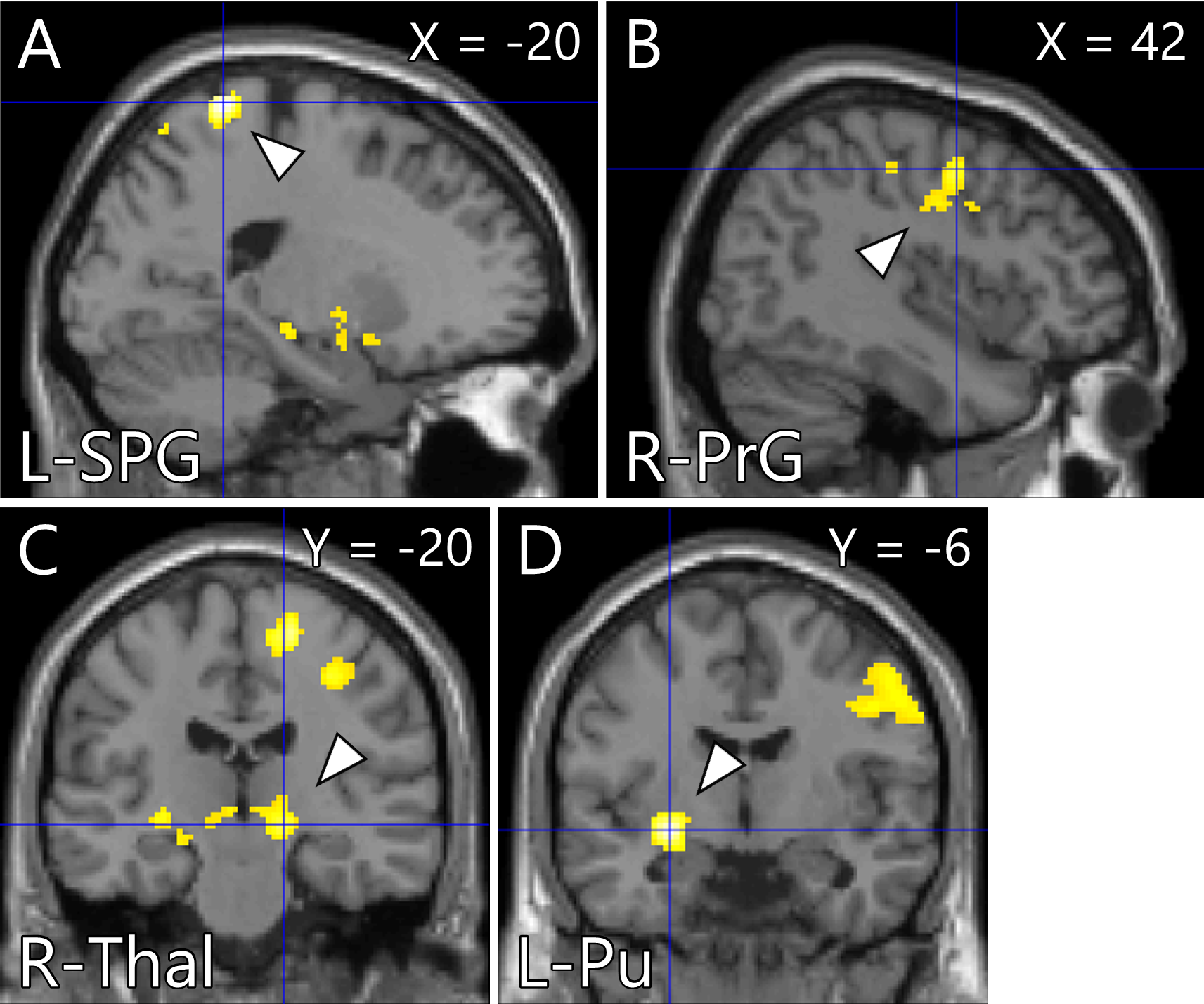
Fig. S2

SUPPLEMENTARY TEXT

Preparation for task stimuli

The authors and 14 healthy Japanese university students (11 men and 3 women, age range, 20–24 years; mean, 21.8 years) created 300 candidate scenario stimuli consisting of three short sentences. All scenarios had to include some characters and be related to a daily social situation. The first two sentences of each scenario denoted an ambiguous outcome. Two types of affective outcome (positive or negative) were prepared for each ambiguous setting.

The candidate scenario stimuli were validated in a preliminary experiment and screened to use in the functional magnetic resonance imaging experiment. A total of 19 healthy Japanese university students (12 men and 7 women, age range, 20–31 years; mean, 21.7 years) participated in the preliminary experiment. Task stimuli were presented on a screen of laptop computers, and participants responded by pressing keypads. Participants conducted the task at their own pace, and all rating and response times were recorded and analyzed. The first two ambiguous sentences were presented, and participants imagined the situations. When the participants had imagined the situation sufficiently clearly, they pressed a space key to move on to the next phase. After the first two sentences were imagined, participants were required to freely expect the subsequent outcome and rate how positive was the imagined outcome on an 8-point Likert scale from 1 (It was very negative) to 8 (It was very positive). Then, a sentence representing a positive outcome was presented, and participants imagined the situations and rated how positive was the presented outcome on an 8-point scale. Moreover, participants rated how vividly they had imagined the scenario on an 8-point Likert scale from 1 (Not at all vividly) to 8 (Very vividly). One session consisted of 100 scenarios, and participants completed in total three sessions at their own pace.

Task stimuli were screened in a manner to satisfy three conditions, “the scenarios could be easily imagined vividly,” “the two initial sentences were affectively ambiguous,” and “the presented outcomes were rated positively.” The scenarios that were not imagined very vividly were excluded based on the vividness ratings (< mean – 1 SD). The top 30 scenarios having large variance (SD) of positive ratings for the expected outcome were evaluated for their affective ambiguity and selected as the assessment task stimuli. We defined a combining score by multiplying the "variance (SD) of the positive ratings for expected outcome" and "score of positive ratings for presented outcome" for each scenario. The top 100 scenarios having high combined score were evaluated for having both affective ambiguity and positive outcomes and selected as the intervention task stimuli.

The periods of the imagining phase and rating phase in the functional magnetic resonance imaging task were set based on the response time in the preliminary experiment. The period of the imagining phase was set at 10 s, which was longer than the mean + 1 SD (4.77 + 3.10 = 7.87 s) of the response time for imagining in the first session of the preliminary experiment. The period of the rating phase was set at 5 s, which was longer than the mean + 1 SD (2.22 + 1.79 = 4.01 s) of the response time for ratings in the first session of the preliminary experiment.

The Fear of Negative Evaluation Scale (FNE)

The FNE consists of 30 true/false items referring to expectation and distress related to negative evaluation from others in social situations (e.g., "I am afraid that others will not approve of me"). Ishikawa *et al*. (1992) translated and standardized the FNE for the Japanese population and reported its sufficient reliability and validity demonstrated by test-retest reliability (r = .76), item-total correlation analysis, and good-poor analysis. Several previous CBM-I studies adopted the FNE for assessment of CBM-I intervention effectiveness (Turner *et al*., 2011; Bowler *et al*., 2012; Hoppitt *et al*., 2014; Khalili-Torghabeh *et al*., 2014; Mobini *et al*., 2014).

fMRI data acquisition and preprocessing

Functional images were acquired using the echo-planar functional images sensitive to blood oxygenation level-dependent contrast (64×64 matrix, TR=2,000 ms, TE=30 ms, FA=80°, FOV=192 mm, 32 slices, 4-mm slice thickness). To acquire a fine structural whole-head image, magnetization-prepared rapid-acquisition gradient-echo images were obtained (240×240 matrix, TR=6.5 ms, TE=3 ms, FA=8°, FOV=240 mm, 162 slices, 1-mm slice thickness).

Preprocessing and statistical analyses for all images were performed using SPM12 (Wellcome Department of Cognitive Neurology, London, UK) implemented in MATLAB (www.mathworks.com). During preprocessing, images were corrected for slice-timing and head motion, spatially normalized to the Montreal Neurological Institute (MNI)–T1 template using the parameter to co-register and normalize the structural image for the MNI–T1 template obtained by the segmentation process for each subject, and smoothed using a Gaussian kernel of 8 mm full width at half maximum.

fMRI data analysis of the assessment task

For the first-level analysis, all images of two sessions (Pre, Post) were included in one model. Contrast images were obtained by the difference between post and pre intervention (Post − Pre) within subjects.

For the second-level analysis, we used two-sample t-tests (assuming unequal variance and independence between groups) of contrast images for the CBM and PN groups from the first-level analysis. To examine the interaction effect between Group and Change in social anxiety (ΔSA), we performed a voxel-wise multiple regression analysis using the mean-centered ΔSA for each group as a covariate of interest.

Analysis for the vividness ratings

Differences between groups in the intervention task were statistically tested by paired-t test and the interaction between time and group in the assessment task was tested by mixed ANOVA. Correlation analysis between the vividness ratings in the intervention task and change (Post − Pre) in interpretation bias or social anxiety scores was conducted for each group.

As a result, the difference between the groups in the intervention task was not significant (*t*(18)=0.11, p=0.91, r=0.03; CBM group: *M*=5.41, 95% CI=[5.01, 5.82]; PN group: *M*=5.38, 95% CI=[4.98, 5.78]). The interaction between time and group in the assessment task were not significant (*F*(1, 36)=2.04, p=0.16, η_p_^2^=0.054). The vividness ratings in the intervention task and amount of change (Post−Pre) in interpretation bias were significantly positively correlated in the cognitive bias modification (CBM) group (r=0.49, p=0.035) but not in the positive-negative (PN) group (r=0.06, p=0.80). The correlation with social anxiety was marginally significant in the CBM group (r=-0.45, p=0.053) but not in the PN group (r=0.09, p=0.71).

Discussion regarding potential confounding factors

The present experimental design could not completely exclude several confounding factors regarding the intervention task that potentially offer alternative explanations for the present results. We collected vividness ratings during the intervention task and could validate that there was no significant difference between the groups regarding imagery vividness (Table 1). However, in addition to vividness, degrees of arousal, familiarity, or movement may also have influenced the differences in brain activities. To countermeasure these concerns, we supplementary analyzed imaging data of the intervention task. The positive and negative trials included in the PN group were modeled as separate regressors and compared with the CBM group using only positive trials in the two-sample t-test. In other words, positive trials that were presented consistently in the CBM group and scrambled with negative trials in the PN group were compared with each other. For example, if we assume that the activity differences in the motor cortex between the CBM and PN groups derived from the differences in degree of virtual body movement between the positive and negative scenarios, the comparison between positive trials in each group should have returned no significant results. As a result, the detected clusters did not markedly differ from previous results and the activity in the somatomotor and somatosensory areas and thalamus remained in the positive-trial comparison (Supplemental Fig. S2, Table S1). Therefore, we speculate that qualitative differences between positive and negative scenarios, which might be confounding, were not sufficient to explain the differences in brain activities in the intervention task. Another possible concern is that changes in mood or emotional states that might have confounded the effectiveness of social anxiety reduction by intervention were not assessed in this study. Previous studies have reported that a change in mood state was not sufficient to induce cognitive bias modification (Salemink and van den Hout, 2010; Standage *et al.*, 2010). Thus, although actually we did not control for the confounding effects of mood states in this study, we expect that the mood states did not play a key role in reducing social anxiety or modifying interpretation bias. However, such potential confounding factors within the experimental and control conditions should be carefully removed to the extent possible in a future study.

**REFERENCES**

Bowler, J. O., Mackintosh, B., Dunn, B. D., Mathews, A., Dalgleish, T., & Hoppitt, L. (2012) A comparison of cognitive bias modification for interpretation and computerized cognitive behavior therapy: effects on anxiety, depression, attentional control, and interpretive bias. *Journal of Consulting and Clinical Psychology*, 80, 1021-33.

Hoppitt, L., Illingworth, J. L., MacLeod, C., Hampshire, A., Dunn, B. D., & Mackintosh, B. (2014) Modifying social anxiety related to a real-life stressor using online cognitive bias modification for interpretation. *Behaviour Research and Therapy*, 52, 45-52.

Ishikawa, R., Sasaki, K., & Fukui, I. (1992) Standardization of Japanese version of FNE and SADS. *Koudou Ryouhou Kenkyu (Jap J Behav Ther)*, 18, 10-7.

Khalili-Torghabeh, S., Fadardi, J. S., Mackintosh, B., Reynolds, S., & Mobini, S. (2014) Effects of a multi-session cognitive bias modification program on interpretative biases and social anxiety symptoms in a sample of Iranian socially-anxious students. *Journal of Experimental Psychopathology*, 5, 514-27.

Mobini, S., Mackintosh, B., Illingworth, J., Gega, L., Langdon, P., & Hoppitt, L. (2014) Effects of standard and explicit cognitive bias modification and computer-administered cognitive-behaviour therapy on cognitive biases and social anxiety. *Journal of Behavior Therapy and Experimental Psychiatry*, 45, 272-9.

Salemink, E., & van den Hout, M. (2010) Trained interpretive bias survives mood change. *Journal of Behavior Therapy and Experimental Psychiatry*, 41, 310-5.

Standage, H., Ashwin, C., & Fox, E. (2010) Is manipulation of mood a critical component of cognitive bias modification procedures? *Behaviour Research and Therapy*, 48, 4-10.

Turner, R., Hoppitt, L., Hodgekins, J., Wilkinson, J., Mackintosh, B., & Fowler, D. (2011) Cognitive bias modification in the treatment of social anxiety in early psychosis: a single case series. *Behavioural and Cognitive Psychotherapy*, 39, 341-7.
